# Effect of Length of Stay on Smoking among Turkish and Eastern European Immigrants in Germany—Interpretation in the Light of the Smoking Epidemic Model and the Acculturation Theory

**DOI:** 10.3390/ijerph121215030

**Published:** 2015-12-15

**Authors:** Katharina Reiss, Reinhard Schunck, Oliver Razum

**Affiliations:** 1 Department of Epidemiology & International Public Health, Bielefeld School of Public Health, Bielefeld University, P.O. Box 100131, Bielefeld 33501, Germany; oliver.razum@uni-bielefeld.de; 2 Aid Information Service on Food, Agriculture, Consumer Protection, Heilsbachstraße 16, Bonn 53123, Germany; 3 Department of Sociology, Bielefeld University, P.O. Box 100131, Bielefeld 33501, Germany; reinhard.schunck@uni-bielefeld.de

**Keywords:** emigrants and immigrants, acculturation, smoking, length of stay, longitudinal studies

## Abstract

*Background*: We analyzed changes in smoking by length of stay among immigrants in Germany and related them to the “smoking epidemic” model and the acculturation theory. *Methods*: We used data from a longitudinal survey (German Socio-economic Panel). Immigrants were identified by country of birth (Turkey: respondents *n* = 828, observations *n* = 3871; Eastern Europe: respondents *n* = 2009, observations *n* = 7202; non-immigrants: respondents *n* = 34,011, observations *n* = 140,701). Smoking status data was available for nine years between 1998 and 2012. Length of stay (LOS, in years) was used as proxy for acculturation. We calculated smoking prevalences, prevalence ratios and a random intercept multilevel logistic regression model. *Results*: With each year spent in Germany, smoking prevalence increases among Turkish women (OR = 1.14 (95%CI = 1.06–1.21)) and slightly decreases among men. Recently immigrated Turkish women smoke less than non-immigrant women (0–5 years: SPR = 0.25 (95%CI = 0.10–0.57)); prevalences converge with increasing LOS (31+ years: SPR = 1.25 (95%CI = 1.06–1.48)). Among Eastern European immigrants no significant changes were apparent. *Conclusions*: Immigrants from Turkey “import” their smoking prevalence from a country which is in the earlier stages of the “smoking epidemic”. With increasing LOS (thus, advancing acculturation), they “move” to the later stages. Anti-smoking interventions should consider different smoking attitudes in Turkey/Germany and need to discourage women from initiating smoking. Future research should also identify reasons for the possible differences between immigrant groups.

## 1. Introduction

Smoking is one of the riskiest health behaviors. It is the leading cause for a variety of cancers and cardiovascular diseases, and it accounts for premature death and causes great economic burden [1,2,3]. According to Lopez *et al.* [4] and Thun *et al.* [5], different countries move through the stages of the “smoking epidemic” at different paces and thus are at different stages in any given moment in time. The “smoking epidemic” is a model with four stages describing trends in smoking over time. Economically developed countries are currently located towards the advanced stages of the epidemic, with a decreasing trend in smoking prevalence among men and women [5,6,7]. Economically less-developed or developing countries tend to be located more towards the early phases of the epidemic with a very high smoking prevalence among men and a low prevalence among women [8,9,10].

Thus, the following question arises: how do immigrants change their smoking behavior if they migrate between countries which are at different stages of the “smoking epidemic”? Do they adapt to the smoking patterns of the host country’s majority population as a result of an acculturation process? Acculturation refers to a process during which values, attitudes, and behaviors of individuals are shaped by interactions with persons of their new social and cultural environment [11,12]. This can encompass unidimensional acculturation, that is, immigrants adapt to the standards of the host country, but it can also encompass a bidimensional acculturation and thus a simultaneous adaptation to some standards of the host country, e.g., in terms of language, while maintaining others of the country of origin, e.g., in terms of health behaviors. Whether or not immigrants maintain their behavior or adapt towards the host country depends on a variety of different factors: the behavior itself (smoking, dietary habits, *etc.*), the immigrant group (e.g., in terms of push and pull factors, group identity *etc.*) and the perception of differences between the population of the country of origin and the host country. An adaptation, for example, might result from an attempt to reduce these perceived differences [11,12,13,14].

Acculturation models are frequently used in health research, with measures ranging from multi-item scales to proxy measures. The most widely used proxy measures are length of stay and proficiency in the main language of the host country [15,16,17]. Studies on acculturation and smoking from the United States revealed that a comparatively high smoking prevalence among less-acculturated immigrants decreases with advancing acculturation, whereas a low smoking prevalence among less-acculturated immigrants increases with advancing acculturation [18,19,20,21].

Germany is one of the main European receiving countries of immigration today. In 2012, 11 million persons or 13.3% of the entire population were immigrants born outside Germany [22]. The overall immigration to Germany decreased between 1991 and 2009 and has been increasing since then (2013: 1.3 million immigrants)—especially in recent times due to a large influx of asylum seekers [23]. Numerically the two largest groups are persons born in Turkey (1.5 million) and persons born in Eastern European countries (among them 2.4 million persons from the former Soviet Union countries). The majority of the latter group are ethnic German repatriates, the so-called resettlers (German: “(Spät-)Aussiedler”).

Locating Germany, Turkey and Russia, the country where the majority of Eastern Europeans emigrate from, according to the stages of the “smoking epidemic” is difficult. However, by comparing the tobacco consumption (g *per capita*) and the smoking prevalence over the last decades, it can be assumed that Germany is positioned more towards the advanced stage and Turkey as well as Russia more towards in earlier phases [6,24,25,26,27,28].

The trends in smoking behavior related to the acculturation process among immigrants in Germany are not yet fully understood. Moreover, due to the latest influx of asylum seekers to Germany, the topic of migration and health will remain of special importance and relevance. Thus, our aim was: (1) to analyze the changes related to an increasing length of stay (as a proxy for acculturation) in smoking prevalence among Turkish and Eastern European immigrant men and women; and (2) to relate possible changes to the stages of the “smoking epidemic” as well as the acculturation theory by comparing smoking prevalences between the two immigrant groups and non-immigrant Germans.

## 2. Methods

### 2.1. Data Source

The data for this study come from the German Socio-economic Panel (SOEP). The SOEP is an ongoing, large-scale, longitudinal survey of households in Germany running since 1984 and being representative of the population in Germany [29]. The SOEP contains two samples which have been explicitly designed to (over)sample ethnic German resettlers and former guest workers (German: “Gastarbeiter”), such as immigrants from Turkey [30]. Thus, it is well suited for the study of immigrants, in particular of those groups this study focuses on. Details on sampling, response and attrition have been published elsewhere [30,31]. New samples are added to the SOEP data at regular intervals to replenish the sample and ensure continuing representativeness of the population in Germany [29,30]. Since the SOEP covers a wide range of topics, including self-reported health behaviour—such as smoking beginning in 1998—it is widely used to study health related outcomes [32,33,34,35]. We used the SOEP data set version 29 which includes data until the year 2012. 

### 2.2. Study Sample

The two numerically largest immigrant groups in Germany, originating from Turkey and Eastern Europe, were identified based on their country of birth (Eastern Europe comprises the former Soviet Union countries, Poland, Romania, Hungary, Bulgaria, the Czech Republic, Albania and Slovakia; ex-Yugoslavian countries were excluded). Thus, only foreign-born persons were included in the analysis. Non-immigrants were persons who themselves and whose parents were born in Germany. We included all respondents aged 17 years and older. Item non-response rates to the study variables are low. Among immigrants, the highest item non-response rates are 4.0% for immigration year and 4.5% for years of education; for non-immigrants 2.7% for years of education. Item non-response rates for all other variables used, including self-reported smoking, are below 1.0%. After the exclusion of cases with missing values (466 for Turkish immigrants, 826 for Eastern European immigrants, and 8060 for non-immigrants), the longitudinal sample comprised 3871 observations (*i.e.*, annual interviews) from 828 Turkish immigrants, 7202 observations from 2009 Eastern European immigrants, and 140,701 observations from 34,011 non-immigrants. The follow up rate in the SOEP is acceptable [31]. In our sample the overall annual attrition rate is 10.6%, it is slightly higher among immigrants (14.1%) than among non-immigrants (10.3%). We conducted multivariate attrition analyses, which revealed that attrition is significantly associated only with marital status and survey year for immigrants. For non-immigrants, attrition is significantly associated with marital status, age, labour force status, and survey year. Our models therefore adjust for these characteristics. Importantly, however, attrition is unrelated to our dependent variable smoking for both immigrants and non-immigrants. Collinearity among the variables in our sample is low: the mean variance inflation index for all modes is below 2.2.

### 2.3. Outcome and Exposure Measures

In the SOEP participants are asked: “Do you currently smoke?” in the years 1998, 1999, 2001, 2002, 2004, 2006, 2008, 2010, and 2012. Those answering “Yes” were classified as smokers. Length of stay (LOS) was calculated as the difference between year of the survey and year of immigration to Germany. Age (≤30, 31–40, 41–50, 51–60, ≥61) and marital status (married, single, other marital status) were included in the analyses, as well as labour force status (working, unemployed, non-working), education (in years), annual net household income (in 1000 Euro) and survey year.

### 2.4. Statistical Analyses

To analyse the trend related to an increasing LOS (aim 1) we performed descriptive and multivariate analyses. First, we calculated smoking prevalence among both immigrant groups by LOS categorized in 5-year-periods. Second, as longitudinal data are hierarchical data—because a given person is measured repeatedly over time—we have estimated a random intercept multilevel logistic regression model. This is a standard method for analyzing longitudinal or clustered data with dichotomous responses [36]. The model is given as:

(1) $$\text{logit }(\text{Pr}\left({\text{y}}_{\text{ij}}\;=\;1|{\text{x}}_{\text{ij}},\zeta_{\text{i}}\right)=\alpha +\beta_{\text{k}}{\text{x}}_{\text{ij}}+\zeta_{\text{i}}$$

where the subscript i denotes person and j denotes measurement occasion, *i.e.*, observation. ${\text{y}}_{\text{ij}}$ is the response variable, indicating if person i is smoking at occasion j, α is the intercept, ${\text{x}}_{\text{ij}}$ a vector of covariates and $\beta_{\text{k}}$ the associated regression weights and $\zeta_{\text{i}}$ are the random effects. Random effects models belong to the class of so called multilevel models, which are also known as mixed models or hierarchical models [36]. It is assumed that $\zeta_{\text{i}}\sim \text{N }\left(0,\psi \right)$ and $\text{Cov }(\zeta_{\text{i}},{\text{x}}_{\text{ij}})=0$. Random intercept model assume that all measurements from a single person share a latent, unobserved random effect ($\zeta_{\text{i}}$), which creates an association in repeated measurements. In our case this means that some respondents have a lower and some a higher probability to smoke.

To compare smoking prevalences between immigrants and non-immigrants (aim 2) we calculated age-standardised prevalence ratios (SPR) and 95% confidence intervals for both immigrant groups. We used an indirect standardisation as it yields more stable results for low number of cases. Non-immigrant men and women served as standard populations, respectively. We weighted the age-specific number of immigrants (who provided information on their smoking status) with the smoking prevalences in the respective age groups among non-immigrants. This resulted in the expected number of smokers among immigrants across all age groups. In a final step the observed number of smokers was divided by the expected number. Since smoking behaviour differs markedly between men and women, all analyses were stratified by gender. Analyses were conducted using Stata 13.1 (College Station, TX, USA).

## 3. Results

The Turkish immigrants were on average 43.1 years old and thus younger than both the Eastern European immigrants (45.6 years) and the non-immigrants (49.6 years). LOS in Germany was on average shorter among immigrants from Eastern Europe than among Turkish immigrants (16.8 years *vs.* 24.3 years). The crude smoking prevalence was highest among Turkish immigrants (39.3%), followed by non-immigrants (28.6%) and Eastern European immigrants (26.6%) (Table 1).

**Figure 1:**
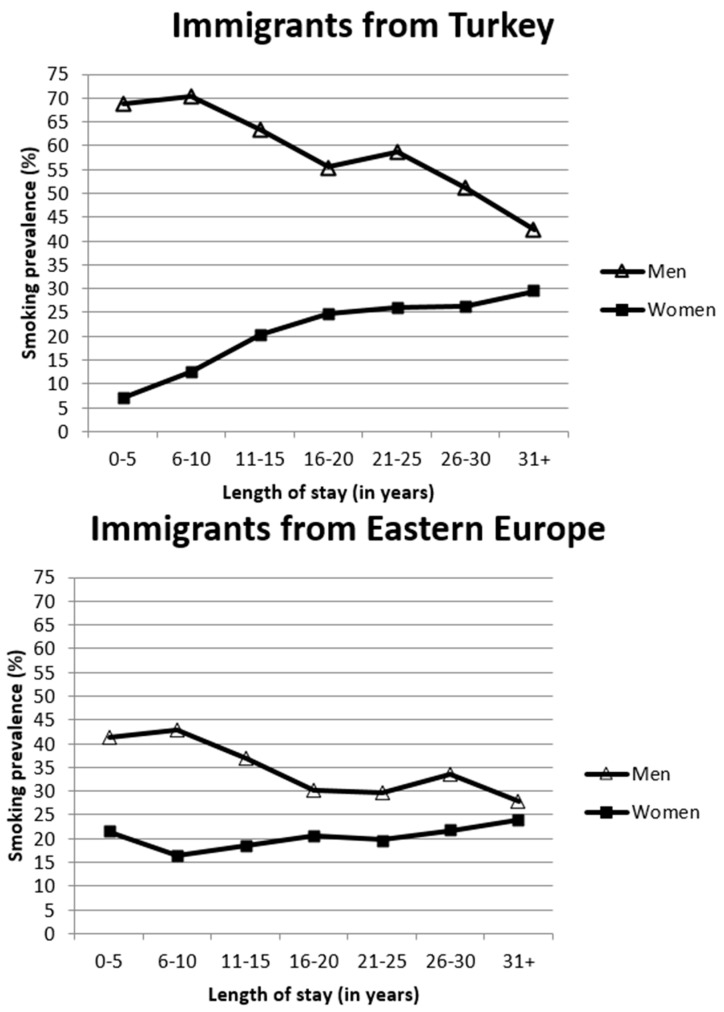
Smoking prevalence by length of stay among immigrants from Turkey and Eastern Europe.

Among Turkish immigrants, the smoking prevalence among men decreases with increasing LOS while it increases among women (Figure 1). As a consequence, the gap between men and women is very large among recent immigrants and diminishes with increasing LOS in Germany. A similar decreasing trend can be observed among men from Eastern Europe; however, a clear increase in smoking prevalence does not apply to women from Eastern Europe.

The multivariate results are displayed in Table 2. For women from Turkey, smoking probability is increasing significantly with LOS in Germany (OR = 1.14 (95%CI = 1.06–1.21)). Thus, the odds of smoking increase by 14% with each additional year spent in Germany. A clear secular time trend between 1998 and 2012 is not discernible for women from Turkey. Additionally, non-working women have a lower smoking probability compared to working women, as do older respondents compared to younger ones. For men from Turkey, the association between LOS and smoking does not reach statistical significance (OR = 0.96 (95%CI = 0.90–1.02)). As in women, smoking probability declines with age. The secular time trend appears to be negative, although this association is not statistically significant for most years. For immigrants from Eastern Europe, there does not seem to be a significant association between smoking probability and LOS, neither for women (OR = 1.03 (95%CI = 0.99–1.08)) nor for men (OR = 0.98 (95%CI = 0.94–1.02)). A significant secular time trend is not found as well. Again, older immigrants are less likely to smoke than younger ones and the annual income is negatively associated with smoking probability. High education (*vs*. low) and non-working labour force status (*vs*. working) decrease the odds of smoking in men from Eastern Europe. Among non-immigrant women, smoking probability increases slightly until the beginning of the last decade and decreases since then. In non-immigrant men the secular time trend is clearly negative. Among non-immigrants there are also negative age effects. Moreover, married individuals display lower odds of smoking compared to singles or those of other marital status, as do non-working individuals compared to working ones. Both education and income significantly decrease the odds of smoking.

**Table 1:** Characteristics of the multivariate study sample (SOEP data set version 29).

Variable		Immigrants from Turkey (*n* = 3871 Observations)		Immigrants from Eastern Europe (*n* = 7202 Observations)		Non-Immigrants (*n* = 140,701 Observations)	
		N	%	N	%	N	%
**Age (in years)**	Up to 30	614	15.9	1508	20.9	21,133	15.0
	31–40	1415	36.5	1528	21.2	25,041	17.8
	41–50	721	18.6	1546	21.5	29,116	20.7
	51–60	565	14.6	1163	16.2	24,551	17.5
	61 and older	556	14.4	1457	20.2	40,860	29.0
		Mean: 43.1 (SD: 13.2; range: 18–86)		Mean: 45.6 (SD: 16.4; range: 17–99)		Mean: 49.6 (SD: 17.0; range: 17–102)	
**Sex**	Male	2035	52.6	3185	44.2	67,096	47.7
	Female	1836	47.4	4017	55.8	73,605	52.3
**Marital Status**	Married	3470	89.6	5318	73.8	90,174	64.1
	Single	176	4.6	1117	15.5	30,261	21.5
	Other marital status	225	5.8	767	10.7	20,266	14.4
**Annual net household income (in 1000 Euro)**		Mean: 28.9 (SD: 16.2; range: 0–177.9)		Mean: 30.0 (SD: 16.8; range: 0–231.4)		Mean: 37.3 (SD: 32.7; range: 0–4282.0)	
**Labour force status**	Working	1908	49.3	4302	59.7	82,512	58.7
	Unemployed	433	11.2	565	7.9	6664	4.7
	Non-working	1530	39.5	2335	32.4	51,525	36.6
**Education (in years)**		Mean: 9.6 (SD: 2.1; range: 7–18)		Mean: 11.4 (SD: 2.3; range: 7–18)		Mean: 12.2 (SD: 2.6; range: 7–18)	
**Length of stay (in years)**	0–5	132	3.4	407	5.6	not applicable	
	6–10	299	7.7	1764	24.5		
	11–15	356	9.2	1944	27.0		
	16–20	464	12.0	1325	18.4		
	21–25	674	17.4	705	9.8		
	26–30	862	22.23	297	4.1		
	31+	1084	28.0	760	10.6		
		Mean: 24.3 (SD: 9.7; range: 1–53)		Mean: 16.8 (SD: 10.7; range: 0–63)			
**Smoking**	No	2348	60.7	5286	73.4	100,454	71.4
	Yes	1523	39.3	1916	26.6	40247	28.6

The comparison between immigrants and non-immigrants reveals that recently immigrated women smoke less than non-immigrant women, irrespective of country of origin (e.g., Turkey (0–5 years): SPR = 0.25 (95%CI = 0.10–0.57)) (Table 3). Among women from Turkey prevalences converge towards those of their counterparts with increasing LOS in Germany; those who reside in Germany for 31 years and longer smoke even more than non-immigrant women (SPR = 1.25 (95%CI = 1.06–1.48)). Eastern European women with a long LOS in Germany do not significantly differ from non-immigrant women in terms of smoking. Contrary to the findings among women, recently immigrated Turkish men smoke more than non-immigrant men (0–5 years: SPR = 1.67 (95%CI = 1.22–2.26)). Those with a long LOS still have a higher smoking prevalence than non-immigrant men, although the difference between both groups seems to become smaller (in terms of a convergence). Among Eastern European men neither a clear difference in smoking prevalence compared to non-immigrant men nor a trend of convergence can be observed.

**Table 2:** Random effects logistic regression model of smoking status among immigrant and non-immigrant participants.

Covariate	Women from Turkey		Men from Turkey		Women from Eastern Europe		Men from Eastern Europe		Non-Immigrant Women		Non-Immigrant Men	
	OR	95% CI	OR	95% CI	OR	95% CI	OR	95% CI	OR	95% CI	OR	95% CI
**Length of stay (in years)**	1.14 ***	(1.06, 1.21)	0.96	(0.90, 1.02)	1.03	(0.99, 1.08)	0.98	(0.94, 1.02)	-	-	-	-
**Up to 30 years**	ref.	-	ref.	-	ref.	-	ref.	-	ref.	-	ref.	-
**31–40 years**	0.45	(0.20, 1.01)	1.89	(0.88, 4.03)	0.70	(0.38, 1.30)	0.86	(0.43, 1.74)	0.83 *	(0.69, 1.00)	0.95	(0.79, 1.15)
**41–50 years**	0.28 *	(0.08, 0.91)	1.07	(0.36, 3.23)	0.50	(0.23, 1.12)	0.56	(0.23, 1.36)	0.56 ***	(0.45, 0.71)	0.62 ***	(0.49, 0.79)
**51–60 years**	0.05 ***	(0.01, 0.21)	0.18 *	(0.04, 0.77)	0.20 ***	(0.08, 0.51)	0.30 *	(0.10, 0.85)	0.23 ***	(0.18, 0.30)	0.24 ***	(0.18, 0.31)
**61 years and older**	0.02 ***	(0.00, 0.11)	0.06 **	(0.01, 0.34)	0.03 ***	(0.01, 0.11)	0.09 ***	(0.03, 0.31)	0.04 ***	(0.03, 0.05)	0.05 ***	(0.04, 0.07)
**Married**	ref.	-	ref.	-	ref.	-	ref.	-	ref.	-	ref.	-
**Single**	0.58	(0.09, 3.68)	0.81	(0.28, 2.35)	3.27 **	(1.52, 7.06)	1.01	(0.42, 2.41)	2.68 ***	(2.19, 3.28)	1.80 ***	(1.47, 2.22)
**Other marital status**	1.64	(0.47, 5.69)	3.16	(0.67, 14.88)	3.08 **	(1.48, 6.41)	2.69	(0.83, 8.72)	1.84 ***	(1.53, 2.22)	2.56 ***	(2.07, 3.18)
**Working**	ref.	-	ref.	-	ref.	-	ref.	-	ref.	-	ref.	-
**Unemployed**	1.04	(0.41, 2.59)	0.83	(0.42, 1.62)	1.36	(0.67, 2.77)	0.72	(0.36, 1.46)	0.86	(0.70, 1.07)	1.48 ***	(1.20, 1.82)
**Non-working**	0.34 **	(0.18, 0.65)	1.24	(0.54, 2.88)	0.65	(0.40, 1.06)	0.43 *	(0.22, 0.83)	0.33 ***	(0.29, 0.38)	0.46 ***	(0.39, 0.53)
**Education (in years)**	1.10	(0.90, 1.35)	0.92	(0.77, 1.09)	0.99	(0.86, 1.12)	0.82 *	(0.70, 0.96)	0.75 ***	(0.72, 0.78)	0.73 ***	(0.71, 0.76)
**Annual net household income (in 1000 Euro)**	1.00	(0.98, 1.02)	1.00	(0.98, 1.01)	0.98 **	(0.96, 0.99)	0.97 ***	(0.95, 0.99)	0.99 ***	(0.99, 1.00)	1.00 ***	(0.99, 1.00)
**Survey year: 1998**	ref.	-	ref.	-	ref.	-	ref.	-	ref.	-	ref.	-
**Survey year: 1999**	1.35	(0.61, 2.95)	1.03	(0.56, 1.89)	1.70	(0.79, 3.66)	1.14	(0.62, 2.11)	1.31 **	(1.09, 1.57)	1.18	(1.00, 1.40)
**Survey year: 2001**	1.72	(0.77, 3.83)	0.96	(0.50, 1.83)	1.23	(0.59, 2.56)	0.84	(0.46, 1.52)	1.11	(0.94, 1.32)	0.95	(0.80, 1.12)
**Survey year: 2002**	3.15 **	(1.37, 7.26)	1.09	(0.56, 2.15)	1.53	(0.73, 3.21)	0.82	(0.44, 1.53)	1.21 *	(1.02, 1.44)	1.00	(0.85, 1.18)
**Survey year: 2004**	2.00	(.82, 4.90)	0.72	(0.34, 1.52)	1.37	(0.64, 2.94)	0.75	(0.39, 1.44)	1.09	(0.92, 1.30)	0.81 *	(0.68, 0.95)
**Survey year: 2006**	3.46 *	(1.33, 9.03)	0.66	(0.28, 1.51)	1.59	(0.73, 3.49)	0.78	(0.38, 1.56)	0.97	(0.81, 1.16)	0.65 ***	(0.55, 0.77)
**Survey year: 2008**	2.08	(0.72, 6.01)	0.33 *	(0.13, 0.87)	1.04	(0.45, 2.39)	0.61	(0.28, 1.32)	0.71 ***	(0.59, 0.86)	0.59 ***	(0.50, 0.71)
**Survey year: 2010**	1.02	(0.31, 3.38)	0.56	(0.19, 1.67)	1.48	(0.62, 3.53)	0.90	(0.39, 2.08)	0.78 *	(0.64, 0.95)	0.57 ***	(0.47, 0.68)
**Survey year: 2012**	0.53	(0.13, 2.20)	0.27 *	(0.08, 0.95)	1.31	(0.54, 3.16)	1.00	(0.41, 2.46)	0.82	(0.67, 1.01)	0.50 ***	(0.42, 0.61)
**Constant**	0.00 ***	(0.00, 0.03)	19.24 **	(2.07, 178.83)	0.01 ***	(0.00, 0.03)	13.17 **	(1.86, 93.04)	0.71	(0.42, 1.21)	6.94 ***	(4.17, 11.55)
**Rho (intraclass correlation)**	0.88		0.87		0.91		0.89		0.94		0.93	
**Log likelihood**	−585.33		−820.62		−1101.46		−1277.19		−21,083.70		−22,342.91	
**Persons**	393		435		1118		891		17,678		16,333	
**Observations**	1836		2035		4017		3185		73,605		67,096	

Source: SOEP data set version 29; * *p* < 0.05, ** *p* < 0.01, *** *p* < 0.001.

**Table 3:** Age-Standardised Prevalence Ratios (SPR) and 95% Confidence Intervals for immigrants by length of stay (SOEP data set version 29).

Variable		Women from Turkey ^1^		Men from Turkey ^2^	
		SPR	95% CI	SPR	95% CI
**Length of stay (in years)**	0–5	**0.25**	(0.10–0.57)	**1.67**	(1.22–2.26)
	6–10	**0.40**	(0.26–0.61)	**1.67**	(1.38–2.02)
	11–15	**0.61**	(0.46–0.82)	**1.49**	(1.20–1.84)
	16–20	0.84	(0.65–1.08)	**1.38**	(1.16–1.64)
	21–25	0.87	(0.71–1.08)	**1.48**	(1.29–1.70)
	26–30	1.02	(0.84–1.23)	**1.49**	(1.31–1.69)
	31+	**1.25**	(1.06–1.48)	**1.38**	(1.23–1.56)
		**Women from Eastern Europe ^1^**		**Men from Eastern Europe ^2^**	
		**SPR**	**95% CI**	**SPR**	**95% CI**
**Length of stay (in years)**	0–5	**0.71**	(0.54–0.94)	1.16	(0.92–1.46)
	6–10	**0.59**	(0.51–0.69)	**1.17**	(1.06–1.30)
	11–15	**0.69**	(0.60–0.79)	1.06	(0.96–1.18)
	16–20	**0.77**	(0.65–0.90)	0.90	(0.78–1.04)
	21–25	**0.75**	(0.60–0.94)	0.95	(0.77–1.17)
	26–30	0.99	(0.72–1.35)	1.24	(0.91–1.70)
	31+	1.14	(0.94–1.40)	1.04	(0.85–1.28)

Numbers in bold print indicate significant results; **^1^** Standard population: non-immigrant women; **^2^** Standard population: non-immigrant men.

## 4. Discussion

Our study has two main findings: firstly, with increasing LOS in Germany, smoking prevalence increases among women from Turkey and converges towards that among non-immigrant women, whereas there is a slight decrease in prevalence among men from Turkey. Secondly, among immigrants from Eastern Europe no significant changes in smoking prevalence are observed.

The trends in smoking related to an increasing LOS were likewise observed in other studies [37,38,39,40,41]. These studies also indicate that the most distinct changes in smoking behaviour occur primarily during the first years in the host country. For immigrants from Turkey we are now able to link this trend to the “smoking epidemic” model and the acculturation theory. Firstly, there is a large gap in smoking prevalence between recently immigrated Turkish men and women, and secondly, there is a higher prevalence among recently immigrated men from Turkey and a lower prevalence among recently immigrated women from Turkey compared to non-immigrant men and women. This indicates that immigrants from Turkey emigrated from a country that is located in the early phases of the “smoking epidemic” to a country that is located in the later phases. The “imported” smoking behaviour among recent immigrants changes with increasing LOS [42]. If we assume that an increasing LOS leads to an advancing acculturation process, a health transition from the earlier to the later stages of the “smoking epidemic” takes place, resulting in an adaptation towards the smoking behaviour of Germany’s majority population [26].

According to the Tobacco Control Scale by Joossens and Raw [43] for the year 2013, Germany is ranked second last out of 34 countries (higher positions indicate a more successful tobacco control policy). Compared to other European countries, Germany was and still is often accused of a delay in acting on tobacco control. It was not until 2007 that smoke-free legislation was enforced for public transport and federal facilities [44,45]. Still, smoking prevalence is clearly decreasing for men and decreasing slightly for women [6]. Thus, immigrant men from Turkey are exposed to a uniform trend in Germany: a lower smoking prevalence (compared to men in Turkey) and an overall decreasing trend among men [6,46]. Women from Turkey, however, are exposed to contradictory forces: the decreasing smoking prevalence among women in Germany and the fact that smoking among women is not a stigma. Although it remains speculative, it seems that the adaptation of the smoking behaviour towards women living in Germany has a higher impact on women from Turkey than the overall decreasing smoking trends [47,48].

Reiss *et al.* [41] found an adaptation towards the smoking patterns of men and women in Germany also among ethnic German resettlers. We did not observe such an adaptation among immigrants from Eastern Europe in the SOEP, most of whom are resettlers. This is surprising since their countries of origin are located more towards the early phases of the “smoking epidemic” (such as Turkey) [49]. It might be that—due to their particular history—resettlers differ from the majority population of their countries of origin. In other words, the smoking patterns in Eastern European countries do not apply to resettlers. This is in line with Kyobutungi *et al.* [50] who found the mortality of resettlers to differ significantly from that of the majority population of their countries of origin. Further research is needed in order to investigate possible differences between immigrant groups in Germany with regard to changes in smoking over time.

This study has several strengths. First, it uses longitudinal instead of cross-sectional data to properly trace a time trend in smoking among the same population(s). Second, it additionally focuses on the “smoking epidemic” and the acculturation process. Linking the “smoking epidemic” to acculturation might help to better understand the smoking behaviour among immigrants as both concepts seem to accompany each other. Third, this study focuses on the two largest immigrant groups in Germany, which allows detecting possible differences in smoking between both groups. Besides its strengths, this study also has limitations. A cohort-effect might partially account for the observed findings regarding LOS. The smoking prevalence of immigrants from Turkey with medium/long LOS in Germany might reflect the prevalence in Turkey at the time of migration. However, the longitudinal nature of the data allows us to control for age (groups), which should, at least partially, account for cohort effects, since we are able to observe different age groups over the same LOS. Moreover, a comparison of smoking prevalence in Turkey in the beginning of the 1990s and in 2011 further indicates that the effect of LOS is unlikely to be a cohort effect. In the 1990s prevalences were 58% (men) and 14% (women). In 2011, smoking is still very common among men, although the prevalence decreased (42%). Among women it remained on a low level (13%) [24]. Thus, the pattern observed in our study among immigrants from Turkey does not reflect the temporal changes in smoking behaviour in their country of origin. Thun *et al.* [5] proposed separate “smoking epidemic” models for men and for women since a trajectory in the sense of the original model was, to date, not observed among women from economically less-developed countries. There are not yet studies clearly assigning Turkey to the stages of the epidemic, and a trajectory in terms of the model is debatable. However, the “smoking epidemic” model seems adequate in order to depict differences between the countries—not necessarily to trace trajectories within the countries. 

One of the major limitations of our study is the use of LOS as a proxy for acculturation. Relying on a proxy measure only does not do justice to the interpretation of acculturation as a complex phenomenon. It is debatable whether: (I) a long LOS corresponds to a high acculturation level and whether (II) a high acculturation level corresponds to only a strong adaptation towards the host country culture. Still, studies using multiple measures of acculturation (both direct measures and proxies) state that, at least in terms of behavioral changes, length of stay is a valid measure of acculturation [51,52,53]. Reiss *et al.* [40], for example, used three different measures of acculturation and observed the most distinct and clear association between length of stay and smoking during pregnancy among women from Turkey. This indicates that length of stay, as one of the most frequently used proxy variables, might be an adequate measure of acculturation—if a study has to rely on proxy measures alone. Finally, it should be borne in mind that the number of men and women from Turkey and Eastern Europe is low in some strata, which might account for the non-significant results in the multivariate analyses.

## 5. Conclusions

We can draw valuable conclusions for public health practice from our findings in spite of some limitations. Practitioners planning interventions need to consider the heterogeneity within the immigrant populations and deliver tailored messages. Most importantly, interventions need to address immigrant women arriving from countries which are located in earlier stages of the “smoking epidemic” and help them to refrain from starting to smoke. According to Poonia [54] it is necessary to involve the respective immigrant communities and to motivate important community representatives to support anti-smoking interventions. Our findings indicate that these key persons should not only be trusted and respected within the communities, as suggested by Poonia [54]. The key persons need to understand the attitudes towards smoking in the countries of origin and the host country, the gender roles and their possible change over time in the host country. Thus, immigrants need to be made aware of the (hidden) social and cultural motivators which act after their immigration to Germany and which are likely to affect their attitude towards smoking. This should help to better reach immigrants with anti-smoking interventions, as well as to better adapt already existing messages to the needs of immigrants. In addition, the overall decreasing trends in smoking among men and women in Germany need to be supported. Comprehensive and ubiquitous non-smoking health messages addressing all segments of the population of Germany are likely to affect also immigrants’ perception of smoking since smoking and acculturation are not only individual but also strong social and group phenomena.
